# Safety of repeated transplantations of neurotrophic factors-secreting human mesenchymal stromal stem cells

**DOI:** 10.1186/2001-1326-3-21

**Published:** 2014-07-10

**Authors:** Yael Gothelf, Natalie Abramov, Adrian Harel, Daniel Offen

**Affiliations:** 1 BrainStorm Cell Therapeutics, POB 10019, Kiryat Aryeh, Petach Tikva 49001, Israel

**Keywords:** Stem cells, Mesenchymal stromal cells, ALS, Repeat injections, Cryopreservation

## Abstract

**Background:**

Therapies based on mesenchymal stem cells (MSC) have been shown to have potential benefit in several clinical studies. We have shown that, using a medium-based approach, MSC can be induced to secrete elevated levels of neurotropic factors, which have been shown to have protective effects in animal models of neurodegenerative diseases.

These cells, designated MSC-NTF cells (Neurotrophic factor-secreting MSC, also known as NurOwn™) derived from the patient's own bone marrow, have been recently used for Phase I/II and Phase IIa clinical studies in patients with Amyotrophic Lateral Sclerosis (ALS). In these studies, ALS patients were subjected to a single administration of autologous MSC-NTF cells. The data from these studies indicate that the single administration of MSC-NTF cells is safe and well tolerated.

In a recently published case report, it was shown that repeated MSC-NTF injections in an ALS patient treated on a compassionate basis were safe and well tolerated [Muscle Nerve 49:455-457, 2014].

**Methods:**

In the current study we studied the toxicity and tolerability of three consecutive intramuscular injections (IM) of cryopreserved human MSC-NTF cells in C57BL/B6 mice to investigate the effect of repeated administration of these cells.

**Results:**

Monitoring of clinical signs and immune reactions showed that repeated injections of the cells did not lead to any serious adverse events. Pathology, histology and blood biochemistry parameters tested were found to be within normal ranges with no sign of tumor formation.

**Conclusions:**

Based on these results we conclude that repeated injections of human MSC-NTF are well tolerated in mice. The results of this study suggest that if the outcomes of additional clinical studies point to the need for repeated treatments, such option can be considered safe.

## Background

We have developed a medium-based protocol for inducing the differentiation of bone marrow-derived multi-potent mesenchymal stromal cells (MSCs) into neurotrophic factor-secreting cells (MSC-NTF cells). These MSC-NTF cells have been developed into a novel cell therapy which aims to target the affected motor neurons via delivery of Neurotrophic Factors (NTF) directly to the site of tissue damage in Amyotrophic Lateral Sclerosis (ALS) patients. In this cell therapy approach, the patients’ bone marrow derived MSCs are propagated *ex-vivo* and induced to secrete elevated levels of NTF such as Glial Cell Line Derived Neurotrophic Factor (GDNF) and Brain-derived neurotrophic factor (BDNF), Vascular Endothelial Growth factor (VEGF) and Hepatocyte Growth factor (HGF). These NTF are delivered to the cell body in the spinal cord via intrathecal (IT) administration, and/or to the motor end plates via intramuscular (IM) administration.

*In-vitro* studies demonstrated that the cell-conditioned medium protects neurons against neurotoxic insults, and *in-vivo* studies have shown that NTF-secreting cells have protective effects in several animal models of neurodegenerative diseases such as Parkinson’s disease, multiple sclerosis, Huntington’s disease and sciatic nerve injury where the transplanted animals showed marked improvements [1-5].

Several models of motor neuron disorders have been used for studying the administration of NTF and investigating the regeneration of axons and functional recovery [6-8]. Although some indications of restoration and recovery of the motor functions were shown, clinical trials of systemic or IT administration of recombinant NTF to patients with motor neuron disorders did not show significant efficacy. It was suggested that this may be the results of NTF short half-life, poor delivery and low concentrations at target sites [9,10]. The use of cellular transplants to deliver NTF, either through their normal release from the transplanted cells or after *in-vitro* manipulations of cells for the overexpression of certain NTF, could provide improved sustained delivery.

In fact, in recent studies, human MSCs genetically engineered to secrete GDNF and VEGF and transplanted into a rat model of ALS, were shown to significantly increase the number of neuromuscular connections and motor neuron cell bodies in the spinal cord at mid stages of the disease, to delay disease progression and increase lifespan [7,8].

One of the main issues concerning cell mediated treatments, especially in chronic diseases, is cell survival. In our previous study using MSC-NTF cells [2] we found, as in other reports [11,12] that cell survival was limited to the range of weeks, although the effect was maintained. Since there appears to be no direct correlation between MSC engraftment and treatment response it is suggested that MSCs mediate their function through a “hit and run” mechanism [13].

Nevertheless since the effect of MSC-NTF appears to be transient in patients [14], repeated administrations may be needed to increase the beneficial effect of MSC-NTF cells. Since the cells are autologous and derived from the patients’ own bone marrow, we developed a cryopreservation process that will allow banking of patients’ cells for repeated use, thus avoiding the need for repeated bone marrow aspiration procedures for harvesting of fresh cells. The present study was aimed to evaluate the overall safety and tolerability of repeated administrations of cryopreserved human MSC-NTF cells in mice. In order to address the possible complications following such treatment, we designed a study to evaluate the applicability and safety of repeated IM injections of MSC-NTF cells in mice. Our data indicated that the mice tolerated the treatment well and that the immune response was minimal.

## Methods

### Preparation and characterization of MSC-NTF cells

Human MSCs were isolated from bone marrow mononuclear cells derived from healthy donors (Lonza). The MSCs were expanded and induced to differentiate into MSC-NTF cells using a medium based approach in which cells were incubated for 72 hours in medium containing 1 mM dibutyryl cyclic AMP (cAMP), 20 ng/ml human Basic Fibroblast Growth Factor (hbFGF), 5 ng/ml human platelet derived growth factor (PDGF-AA), and 50 ng/ml human Heregulin β1. MSC-NTF cells deriving from fresh MSC were used for transplantation of the first group of mice.

For all successive transplantations MSC were cryopreserved in 10% Dimethyl sulfoxide (DMSO) in growth medium using a controlled rate freezer (Kryo 360–3.3, Planer Plc UK). After thawing, the MSC were expanded and induced to differentiate into MSC-NTF cells.

MSC and MSC-NTF cells are characterized by phenotypic analyses of cell surface antigens by flow cytometry. As recommended by the International Society for Cellular Therapy (ISCT) [15], human MSC should be characterized by expression of CD105, CD73 and CD90 surface markers. To confirm the purity of the cell population and to exclude the presence of hematopoietic cell contamination, these cells should lack expression of CD3, CD14, CD19, CD34, CD45, and HLA-DR as determined by flow cytometry. MSC-NTF cells are also tested for secretion of neurotrophic factors into the culture supernatant by an ELISA assay.

MSC-NTF cells were transplanted at a concentration of 1 × 10^6^ cells/100 μl in RPMI 1640 medium. Cells were subjected to a gentle short manual mixing prior to each withdrawal of cell suspension into a syringe.

### Mice

In this study we used a total of 20 Male and 20 Female C57BL/6JOlaHsd (Harlan Israel) mice, nine to ten weeks of age. Mice were allowed free access to drinking water, supplied to each cage via polyethylene bottles with stainless steel sipper tubes. The water was filtered (0.1-micron filter), chlorinated, and acidified. During the acclimation period and throughout the entire study duration, animals were housed within a limited access rodent facility and kept in groups of maximum five animals in polypropylene cages (36.5 × 20.7 × 14.0 cm) that were fitted with solid bottoms and filled with wood shavings as bedding material (7093 Harlan Teklad Shredded Aspen). The mice were allowed a six-day acclimation period to facility conditions (20°C–24°C, 30%–70% relative humidity, and a twelve-hour light/dark cycle) prior to inclusion in the study. Animal care and administration of MSC-NTF cells were conducted at a GLP-certified site (Harlan Biotech Israel Ltd., Rehovot, Israel), and approved by the National Council for Animal Experimentation.

### Study design

To assess the potential toxic effects of the MSC-NTF cells, the mice received either single or repeated IM injections to the quadriceps femoris muscles. The first group (#1) of 5 male and 5 female mice was treated with a single IM injection at a single dose. The second group (#2) of 5 male and 5 female mice was injected with 2 successive IM injections with a 4 week interval, and the third group (#3) of 5 male and 5 female mice was injected with 3 successive IM injections at 4 week intervals. An additional group (#4), was injected with 3 successive IM injections at 4 week intervals of RPMI 1640 medium, and served as the vehicle control group. The animals were injected in both right and left lateral thigh muscles via a 25G needle attached to a 1 ml syringe. The MSC-NTF cells, 1 × 10^6^ cells/animal, were injected at the constant dose volume of 100 μl/animal/dosing session, 50 μl/site. The vehicle control group was subjected to identical experimental conditions (Table 1).

**Table 1:** Experimental study design

		**Treatment**			
**Group no.**	**No. of animals per group**	**Test material**	**Dose (cells/animal)**	**Frequency and route**	**Study period**
1	10 (5 males and 5 females)	*MSC-NTF cells*	1 × 10^6^	1 × IM Injection	4 weeks
2	10 (5 males and 5 females)	*MSC-NTF cells*	1 × 10^6^	2 × IM Injections	8 weeks
3	10 (5 males and 5 females)	*MSC-NTF cells*	1 × 10^6^	3 × IM Injections	12 weeks
4	10 (5 males and 5 females)	*Vehicle Control*	0	3 × IM Injections	12 weeks

We selected IM injections as the route of administration in this study, since it represents one of the routes used in our clinical studies. The cell dose used in this study is 50-fold the currently used clinical dose.

We have previously shown that repeated transplantation was safe in compassionate use patients re-injected with MSC-NTF after several months [14]. However, since the positive clinical effect of the treatment was sustained for only about 2–3 months, we sought to explore the possibility of administering repeat injections at shorter intervals. Therefore, this study was aimed to confirm that re-treatments at 4-week intervals could be safely tolerated, while also taking into consideration a minimal recovery time from any possible inflammation that may have been caused in the mice by the previous injection.

## Examinations and observations

### Clinical examination

Individual clinical examinations were carried out immediately post-dosing, and up to the first 30 minutes, and at least twice more during the first 2 hours of each dosing session. Observations included changes in skin, fur, eyes, mucous membranes, occurrence of secretions and excretions (e.g. diarrhea) and autonomic activity (e.g. lacrimation, salivation, piloerection, unusual respiratory pattern). Changes in gait, posture and response to handling, as well as the presence of bizarre behavior, tremors, convulsions, sleep & coma were also followed and recorded. Thereafter and through the entire observation period, cage side clinical examinations were carried out and recorded once daily.

#### Body weight

Individual body weights were determined at randomization/animal receipt procedure, shortly before each administration of cells or vehicle control (dosing day), and thereafter twice weekly, and prior to the respective scheduled termination.

#### Cytokine profile assay

Blood samples of approximately 10% of the total blood volume of each animal were collected. Whole blood was individually collected by retro-orbital sinus bleeding under light CO_2_ anesthesia. Blood samples were taken at 2, 4 and 24 hours post dosing; at 2 hours post the second injection, and at 4 hours post the third injection.

Cytokine profile assay was performed using BMS820FF Mouse Th1/Th2 10plex Ready-to-Use kit for FlowCytomix Multiplex, single well per serum sample for the detection of the following cytokines: GM-CSF, IFN-γ, IL-1α, IL-2, IL-4, IL-5, IL-6, IL-10, IL-17 and TNF-α.

#### Food consumption

Measurements of food consumption were initially carried out during the acclimation period (prior to the first dosing session), followed by weekly measurements throughout the entire observation period. The last food consumption measurement was carried out prior to the respective scheduled termination.

#### Clinical pathology

Hematology and Biochemistry parameters were measured in all animals prior to their respective scheduled termination. Blood samples (approximately 100 μl whole blood collected in EDTA-coated tubes for hematology, and approximately 180 μl serum collected in non-coated tubes for biochemistry) were obtained by retro-orbital sinus bleeding under light CO_2_ anesthesia. Blood samples were analyzed at the American Medical Laboratories (Israel) Ltd. The standard biochemistry parameters were tested using ROCHE-HITACHI/MODULAR P-800 and the hematology standard parameters were analyzed by the Sysmex K × 21 system.

#### Necropsy procedures and macroscopic examination

All animals were subjected to a fully detailed necropsy and gross pathological examination following their respective scheduled terminations. The examination included the external body surface, all orifices, cranial, thoracic and abdominal cavities and their contents and any abnormality or gross pathological changes observed in tissues and/or organs. The following organs/tissues were collected and fixed in 10% neutral buffered formalin (approximately 4% formaldehyde solution) and analyzed by Patho-Lab Diagnostics Ltd.: heart, kidneys, liver, lungs, spleen, brain, inguinal, lymph nodes injection sites (thigh muscle) and thymus.

Histopathological examination: tissues were trimmed, embedded in paraffin, sectioned at approximately 5 microns thickness and stained with Hematoxylin & Eosin (H&E). The injection site (right and left thigh musculature) was trimmed in the middle and about 2 mm proximal and distal to the middle section. The histological processing examinations were carried out by Dr. Abraham Nyska, Israel.

### Data evaluation

Statistical analysis of the following quantitative parameters was confined to a comparison between results obtained for animals that received three repeated IM injections of the MSC-NTF cells and the animals that received the vehicle control. The parameters that were compared were: clinical pathology, organ weight, organ weight to body weight ratio, body weight and percentage change in body weight. Analyses were performed by the following methods in order to determine significance of treatment effects: Software: GraphPad Instat® - Version 3.02 (Statistical Method: 1-Way ANOVA; Unpaired *T* Test), Microsoft® Excel 2000.

## Results

### MSC and MSC-NTF cells characterization

MSC before differentiation were found to express > 95% CD105, CD73 and CD90 surface markers, and to lack expression of CD3, CD14, CD19, CD34, CD45, and HLA-DR (<2%), as determined by flow cytometry. After differentiation, MSC-NTF cells had the same phenotype as MSC with the exception of CD105, which was expressed on about 77% of the cells at the end of differentiation. CD44, another characteristic marker of MSC, was also found to be expressed on > 95% of both the MSC and MSC-NTF cells.

BDNF and GDNF specific productivity of MSC-NTF cells was also shown to be secreted about two and five times higher, respectively, as compared to MSC cells of the same donor (Table 2).

**Table 2:** Neurotrophic factor secretion

	**MSC**	**MSC-NTF**	**Fold induction**
BDNF (pg/10^6^ cells)	827 ± 128	1640 ± 125	1.98
GDNF (pg/10^6^ cells)	161 ± 10	746 ± 68	4.6

### Clinical observation

No mortality as a result of treatment occurred in any of the animals prior to the scheduled study termination. No clinical changes were noted in any of the MSC-NTF treated animals, nor in any of the vehicle control-treated animals throughout the entire study period.

### Body weight

Body weight gain was noted in all animals at all termination time points (i.e. 28, 56 and 84 days post treatment commencement). Mean body weight and percentage change in body weight of groups treated with either a single or repeated injections of the cells were found to be similar to those of the Vehicle Control-treated group (Table 2).

### Organ weights

There was no statistically significant difference in the mean organ weight to body weight ratio between the 3× MSC-NTF treated group (Group No. 3) vs. the Vehicle Control treated group (Group No. 4, Table 3).

**Table 3:** Body weight and selected organ weights of male and female C57BL/6 J mice following single or repeated injections of MSC-NTF cells or the vehicle control

**Treatment**				
	**4-week study**	**8-week study**	**12-week study**	
**Parameter**	***MSC-NTF*** **cells single injection**	***MSC-NTF*** **cells 2 injections**	***MSC-NTF*** **cells 3 injections**	**Vehicle 3 injections**
Males				
Terminal body weight (g)	26.2 (0.73)	27.4 (1.21)	30.3 (1.63)	33.6 (2.78)
Organ weight (g)				
Brain	0.463 (0.012)	0.447 (0.015)	0.468 (0.018)	0.454 (0.03)
Liver	1.437 (0.12)	1.431 (0.114)	1.515 (0.239)	1.828 (0.214)
Lungs	0.188 (0.044)	0.205 (0.022)	0.219 (0.036)	0.205 (0.036)
Heart	0.141 (0.01)	0.154 (0.009)	0.178 (0.041)	0.175 (0.013)
Spleen	0.068 (0.019)	0.08 (0.019)	0.093 (0.019)	0.094 (0.022)
Organ weight to body weight ratio (mg organ weight/g body wt)				
Brain	17.69 (0.85)	16.29 (0.52)	15.45 (0.98)	13.62 (1.7)
Liver	54.77 (3.66)	52.15 (3.44)	49.88 (6.85)	54.38 (1.99)
Lungs	7.16 (1.51)	7.48 (0.94)	7.24 (1.34)	6.16 (1.28)
Heart	5.39 (0.36)	5.64 (0.42)	5.86 (1.22)	5.23 (0.43)
Spleen	2.58 (0.67)	2.95 (0.79)	3.06 (0.6)	2.84 (0.82)
Females				
Terminal body weight (g)	21.9 (1.07)	21.3 (0.90)	23.5 (1.69)	22.7 (0.78)
Organ weight (g)				
Brain	0.443 (0.016)	0.444 (0.026)	0.474 (0.008)	0.462 (0.017)
Liver	1.078 (0.1)	1.051 (0.071)	1.166 (0.124)	1.080 (0.09)
Lungs	0.195 (0.035)	0.167 (0.02)	0.182 (0.034)	0.194 (0.021)
Heart	0.124 (0.012)	0.144 (0.008)	0.147^*^ (0.01)	0.131 (0.011)
Spleen	0.088 (0.013)	0.076 (0.006)	0.112 (0.039)	0.097 (0.024)
Organ weight to body weight ratio (mg organ weight/g body wt)				
Brain	20.19 (0.38)	20.85 (1.88)	20.23 (1.33)	20.36 (0.44)
Liver	49.05 (2.19)	49.24 (2.09)	49.56 (3.72)	47.52 (2.77)
Lungs	8.84 (1.39)	7.84 (1.0)	7.72 (1.24)	8.56 (0.92)
Heart	5.67 (0.37)	6.73 (0.42)	6.28 (0.35)	5.78 (0.55)
Spleen	4.02 (0.38)	3.55 (0.25)	4.82 (1.88)	4.28 (0.94)

Note: Values are mean (*SD).*

^*^Indicates statistical significance at *p ≤ 0.05.*

### Food consumption

Mean group food consumption values (gram/animal/group) throughout all measuring sessions were within the expected range for mice and there were no significant differences between the various MSC-NTF cells-treated and the Vehicle Control group.

### Cytokine profile assay

**Figure 1:**
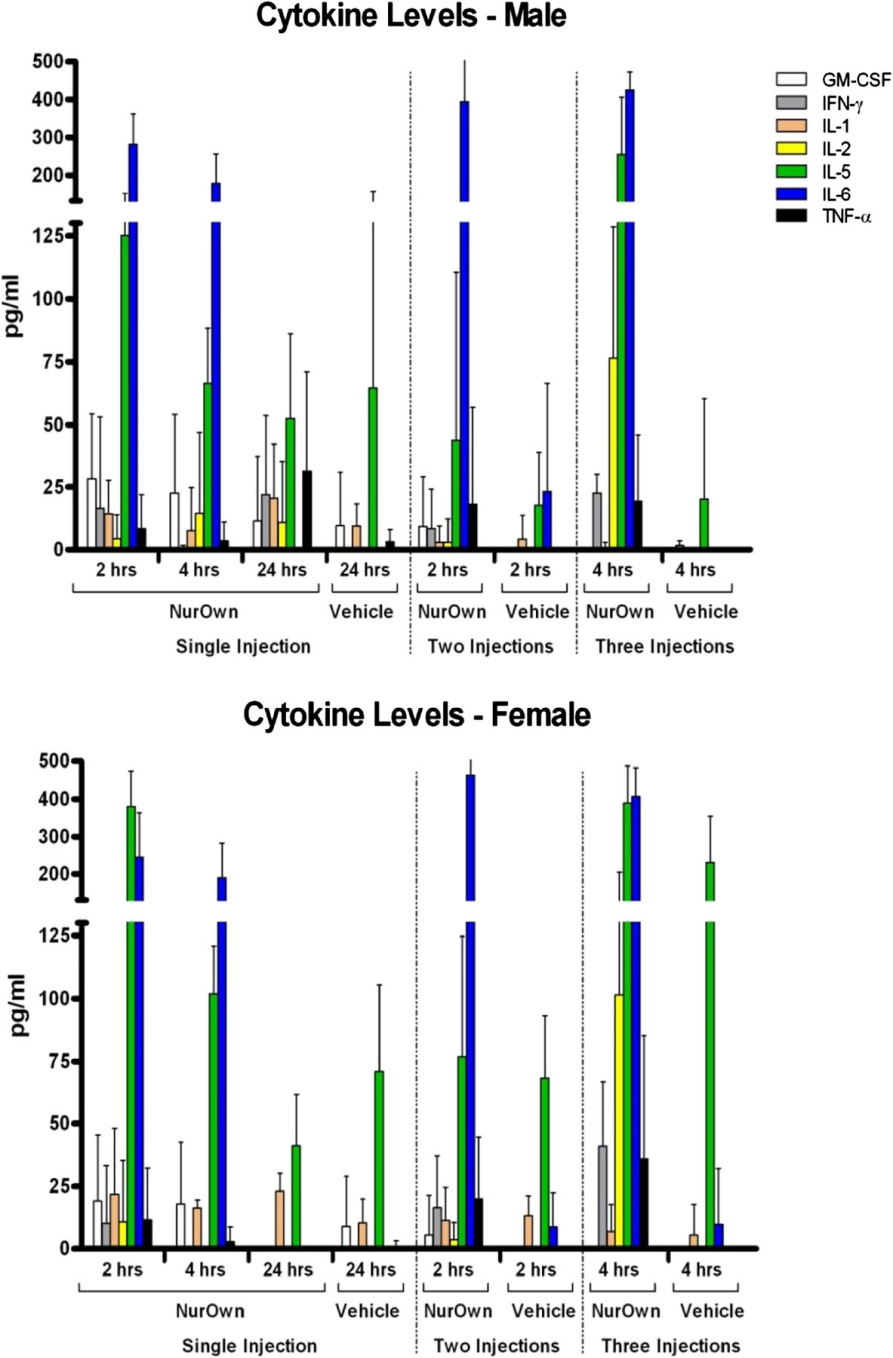
Levels of selected cytokines of male and female C57BL/6 J Mice following single or repeated injections of MSC-NTF cells or the vehicle control.

The ELISA assay was chosen for cytokine profiling of the serum samples in order to detect the levels of the actual proteins secreted.Mean group cytokines secretion levels of groups treated with either a single or repeated injections of the MSC-NTF cells were comparable to those of the Vehicle Control treated group. Although mean group values of IFN-γ, IL-6 and TNF-α of groups treated with repeated IM injections of the MSC-NTF cells were found to be slightly higher than those of the Vehicle Control treated group, those differences were quite small when compared to results obtained using the immunostimulatory compound LPS. The latter induced a biological response in which secretion levels of the cytokines were elevated by 4–5 times, and correlated to clinical changes (Harlan Biotech Israel, Historical Data – Reference Controls, not shown, Figure 1).

A significant difference in secretion of some of the cytokines was identified: IL-1α increased in females *vs* vehicle control group (p < 0.05) after the first injection, IL-6 increased in both males and females after the second injection (p < 0.001), IL-5 and IFN-γ increased in males (p < 0.05 and p < 0.01 respectively) and IL-6 was found to be increased at statistically significant levels in females (p < 0.001) after the third injection.

However since the elevation of the aforementioned cytokines following a single or repeated injections of MSC-NTF cells appeared to be minimal and transient, and did not correlate to clinical changes, it may be concluded that these changes do not indicate a significant biological response.

It is interesting to note that there was no increase in the key regulators of the humoral immune response, IL-4 and IL-10, which would be an indication of a Th2 response, in any of the treatment groups throughout the entire study period.

IL-4 mediates its function by binding to receptors expressed on target cells such as T lymphocytes, and macrophages. In B cells, IL-4 promotes immunological class switching to IgE and IgG1 isotypes, and upregulates Major Histocompatibility Complex (MHC) Class II and CD23 expression. It can promote survival, growth, and differentiation of both T and B lymphocytes, mast cells, and endothelial cells. In addition, IL-4 can inhibit the production of TNF, IL-1, and IL-6 by macrophages.

In addition, IL-4 promotes alternative activation of macrophages into M2 cells and inhibits classical activation of macrophages into M1 cells. An increase in repair macrophages (M2) is coupled with secretion of IL-10 and TGF-β, that result in a diminution of pathological inflammation [16]. IL-10 is a pleiotropic cytokine playing an important role as a regulator of lymphoid and myeloid cell function. In addition, IL-10 participates in regulating proliferation and differentiation of B-cells, mast cells and thymocytes. In parallel, IL-10 inhibits the production of inflammatory mediators from activated macrophages and dendritic cells, probably by STAT3 activation [17].

The fact that our treatment did not induce significant secretion of IL-4 and IL-10 indicates that the repeated injections did not elicit an immunological or inflammatory reaction.

### Hematology and biochemistry

No treatment-related changes in mean group hematology & biochemistry values were noted at 28 days post (last) injection in both the single injection group or in the repeated injections groups. The relatively comparable values in all treated groups suggests a lack of dose-dependent changes and a lack of consistent correlation with other pathological findings (e.g. histopathological or gross pathological findings), thereby implying an absence of target organ effects.

All of the statistically significant differences reported in hematology and biochemistry analyses were found to be within the normal range for the reference values of mice (Tables 4 and 5).

**Table 4:** Selected hematology and biochemistry analysis of male C57BL/6 J mice following single or repeated injections of MSC-NTF cells or the vehicle control

**Treatment**				
	**4-week study**	**8-week study**	**12-week study**	
**Parameter**	***MSC-NTF*** **cells single injection**	***MSC-NTF*** **cells 2 injections**	***MSC-NTF*** **cells 3 injections**	**Vehicle 3 injections**
No of animals analyzed	5	5	5	4
White blood cells (×10^3^/μL)	10.4 (1.9)	11.6 (4.0)	12.2^*^ (1.8)	9.9 (0.4)
Red blood cells (×10^6^/μL)	9.82 (0.34)	9.24 (2.6)	9.72 (1.75)	9.27 (0.31)
Hemoglobin (g/dL)	14.8 (0.1)	16.3 (2.0)	15.0 (2.9)	14.5 (0.4)
HCT (%)	50.7 (1.2)	47.1 (14.0)	48.1 (9.2)	47.9 (1.4)
MCV (U3)	51.6 (1.2)	50.8 (1.2)	49.5^**^ (1.1)	51.7 (0.5)
Creatinine (mg/dL)	0.22 (0.02)	0.16 (0.04)	0.17 (0.03)	0.22 (0.05)
Urea (mg/dL)	51.6 (5.3)	46.0 (8.0)	53.0 (11.1)	47.4 (4.1)
Total Protein (g/dL)	5.81 (0.17)	5.75 (0.16)	5.72 (0.2)	5.79 (0.17)
AST (IU/L)	286 (250)	192 (80)	180 (62)	157 (104)
ALT (IU/L)	502 (430)	163 (69)	197 (92)	87 (47)
ALP (IU/L)	124 (14)	89 (7)	87 (3)	95 (25)
Bilirubin (mg/dL)	0.07 (0.02)	0.08 (0.01)	0.08 (0.05)	0.07 (0.01)
Cholesterol (mg/dL)	138 (3)	144 (12)	133 (26)	153 (13)
TRIG (mg/dL)	137 (6)	65 (14)	70 (27)	68 (6)
Phosphorus (mg/dL)	10.6 (0.8)	11.4 (0.7)	10.7 (1.6)	11.2 (0.8)
LDH (IU/L)	1874 (1300)	906 (197)	1000 (464)	735 (331)

Note: Values are mean (*SD).*

^*^Indicates statistical significance at *p ≤ 0.05.*

^**^Indicates statistical significance at *p ≤ 0.01.*

**Table 5:** Selected hematology and biochemistry analysis of female C57BL/6 J mice following single or repeated injections of MSC-NTF cells or the vehicle control

**Treatment**				
	**4-week study**	**8-week study**	**12-week study**	
**Parameter**	**MSC-NTF cells single injection**	**MSC-NTF cells 2 injections**	**MSC-NTF cells 3 injections**	**Vehicle 3 injections**
No of animals analyzed	5	5	5	5
White blood cells (×10^3^/μL)	11.1 (2.4)	8.5 (1.9)	9.2 (3.0)	9.0 (1.6)
Red blood cells (×10^6^/μL)	9.74 (0.31)	9.65 (0.41)	8.37^*^ (1.66)	10.97 (0.81)
Hemoglobin (g/dL)	14.7 (0.3)	15.1 (0.5)	12.9^*^ (3.1)	17.0 (1.4)
HCT (%)	49.3 (1.5)	49.4 (2.1)	41.9^*^ (8.1)	55.4 (4.5)
MCV (U3)	50.6 (0.9)	51.2 (0.8)	50.1 (1.1)	50.5 (0.7)
Creatinine (mg/dL)	0.22 (0.0)	0.21 (0.04)	0.22 (0.03)	0.21 (0.04)
Urea (mg/dL)	44.0 (2.3)	33.4 (4.3)	45.1 (4.6)	48.0 (5.2)
Total Protein (g/dL)	5.61 (0.12)	5.94 (0.17)	5.81 (0.35)	5.63 (0.12)
AST (IU/L)	224 (140)	186 (46)	109 (38)	95 (28)
ALT (IU/L)	288 (154)	79 (42)	69 (27)	46 (11)
ALP (IU/L)	161 (7)	146 (9)	117 (24)	124 (17)
Bilirubin (mg/dL)	0.11 (0.03)	0.09 (0.01)	0.07 (0.01)	0.08 (0.01)
Cholesterol (mg/dL)	101 (9)	111 (12)	96 (20)	103 (13)
TRIG (mg/dL)	126 (6)	54 (9)	58 (14)	58 (3)
Phosphorus (mg/dL)	10.4 (0.8)	10.7 (0.9)	10.7 (1.4)	10.8 (1.3)
LDH (IU/L)	921 (450)	784 (163)	693^*^ (142)	461 (86)

Note: Values are mean (*SD).*

^*^Indicates statistical significance at *p ≤ 0.05.*

### Macroscopic and histopathological findings

**Figure 2:**
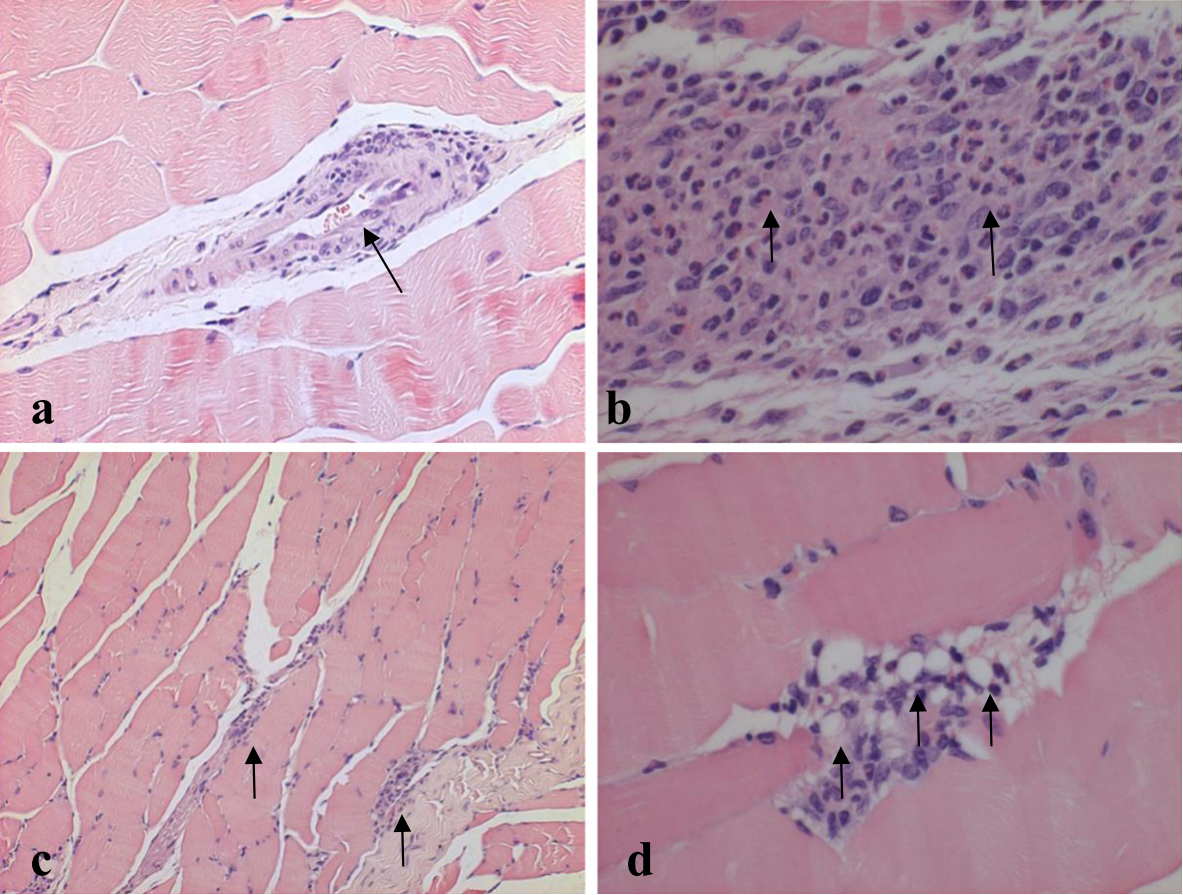
**Sections of the injection site. (a)** blood vessel, minimal subchronic inflammation,. H&E × 100, **(b)** Myofibers - subchronic inflammation, mild. The arrows indicate the presence of polymorphonuclear cells, mixed with other mononuclear cells (i.e., cells having round nucleus and abundant eosinophilic cytoplasm, lymphocytes and plasma cells). H&E, X400. **(c)** Myofibers - subchronic inflammation, minimal. H&E, X100, **(d)** Myofibers - subchronic inflammation, minimal. The arrows indicate the presence of intracellular vacuoles. H&E, X400.

No gross pathological findings related to treatment were evident at necropsy in any of the animals following the scheduled termination. Treatment-related histopathological findings were confined to the injection site of almost all MSC-NTF cells’ treated animals. The range and severity of those findings were mostly of minimal severity and relatively comparable in all MSC-NTF cells’ treated groups. The most consistent injection site lesions observed involved multifocal myofiber subchronic inflammation and presence of intracellular vacuoles at the site of inflammation. Neither tumors nor treatment-related lesions were noted in any of the other evaluated organs (Figure 2, Table 6).

**Table 6:** Histopathological findings in male and female C57BL/6 J mice following single or repeated injections of MSC-NTF cells or the vehicle control

**Histopathological findings mean severity (Number affected/Total number of animals)**				
	**4-week study**	**8-week study**	**12-week study**	
**Organ/tissue**	**MSC-NTF cells single injection**	**MSC-NTF cells 2 injections**	**MSC-NTF cells 3 injections**	**Vehicle 3 injections**
Males				
Injection sites – thigh muscles				
Blood vessel – inflammation subchronic, focal	0.2 (1/5)	0.0 (5/5)	0.0 (5/5)	0.0 (4/4)
Myofiber subchronic inflammation, multifocal	0.6 (2/5)	1.0 (5/5)	1.0 (5/5)	0.0 (4/4)
Presence of intracellular vacuoles at the site of inflammation	0.0 (5/5)	0.6 (3/5)	0.8 (4/5)	0.0 (4/4)
Presence of multinucleated giant cells at the site of inflammation	0.0 (5/5)	0.2 (1/5)	0.2 (1/5)	0.0 (4/4)
Interstitium subchronic inflammation, multifocal	0.2 (1/5)	0.0 (5/5)	0.0 (5/5)	0.0 (4/4)
Interstitium –fibrosis, multifocal	0.2 (1/5)	0.0 (5/5)	0.0 (5/5)	0.0 (4/4)
Females				
Injection sites – thigh muscles				
Myofiber subchronic inflammation, multifocal	1/0 (5/5)	1.0 (5/5)	1.0 (5/5)	0.0 (5/5)
Presence of intracellular vacuoles at the site of inflammation	0.8 (4/5)	0.2 (1/5)	0.8 (4/5)	0.0 (5/5)
Presence of multinucleated giant cells at the site of inflammation	0.2 (1/5)	0.2 (1/5)	0.4 (2/5)	0.0 (5/5)
Granuloma-foreign body, focal	0.4 (2/5)	0.0 (5/5)	0.0 (5/5)	0.0 (5/5)

## Discussion

ALS is a complex disease and cannot be explained by the occurrence of a single event or the disturbance of a single gene or protein. The disease is likely to be the result of a multi-step process, ultimately leading to motor neuron degeneration and clinical symptoms. One of the mechanisms that have been suggested is the “dying-back” pattern that might lead to the progressive loss of motor neurons in ALS [18]. Based on the dying-back hypothesis the muscles are targeted for therapy in ALS patients.

Our preliminary data from a Phase I/II study in ALS patients using our MSC-NTF cells showed that both IT and IM injections are safe [14]. These results are in agreement with several other clinical studies that reported that MSCs derived from different tissues including bone marrow and umbilical cord have shown therapeutic potentials in clinical studies without significant adverse events [19-21]. Moreover, IT MSC transplantation and even repeated intraspinal injection of MSC in ALS patients was found to be safe [22-24].

Since the autologous MSC and MSC-NTF cells are propagated *ex-vivo* before being back-transplanted into the patient, it is necessary to confirm that repeated MSC-NTF cells transplantation does not pose any risk to the patient. The main concern in repeated cell injections is a possible immune response after the first exposure of the mouse to human cells. To follow these immune reactions we used non-immunosuppressed mice and measured the release of Th1/Th2 cytokines.

Th1/Th2 cytokines (also known as Type-1 and Type-2 cytokines) refer to a pattern of cytokines secreted by two different subpopulations of CD4+ T-cells that determine the outcome of an antigenic response toward humoral or cell-mediated immunity. These cytokines are also secreted by other cells like such as CD8 (+) T-cells, monocytes, natural killer cells and B-cells [25]. We measured the profile of Type-1 cytokines including IL-2, IFN-γ, IL-12 and TNF- α, and Type-2 cytokines including IL-4, IL-5, IL-6, IL-10 and IL-13, following a single or repeated injections of MSC-NTF cells. We found transient elevation in IL-5 and IL-6 and to a lower extent IL-2 were elevated after the first cell injection however comparable changes were observed after repeated injections. The other cytokines showed very low or undetectable response.

It is interesting to note that the key regulators of the humoral immune response IL-4 and IL-10 were undetectable before or after the injections.

IL-4 mediates its function by binding to receptors expressed on target cells such as T lymphocytes, and macrophages. On B cells, IL-4 promotes immunological class switching to IgE and IgG1 isotypes and upregulates Major Histocompatibility Complex (MHC) class II and CD23 expression. It can promote survival, growth, and differentiation of both T and B lymphocytes, mast cells, and endothelial cells. In addition, IL-4 can inhibit the production of TNF, IL-1, and IL-6 by macrophages. IL-10 is a pleiotropic cytokine playing an important role as a regulator of lymphoid and myeloid cell function. In addition, IL-10 participates in regulating proliferation and differentiation of B-cells, mast cells and thymocytes. The fact that our treatment didn’t induce significant secretion of IL-4 and IL-10, key regulators in adaptive immunity, indicates that the repeated injections do not elicit an immunological reaction.

Histopathological evaluation revealed neither tumors nor pathological findings in any major organ. Though a treatment-related local effect was noted, the repeated injections were not associated with increased severity of the lesions or any systemic adverse effect. This finding supports the safety of repeated treatments with MSC-NTF.

In a similar study, using human umbilical cord mesenchymal stem cells in cynomolgus monkeys with repeated intravenous injection once every 2 weeks, for 6 weeks, it was demonstrated that no stem cells transplantation-related toxicity was found [26]. Furthermore we have recently shown that repeated MSC-NTF injections in an ALS patient treated on a compassionate basis were safe and well tolerated [27].

In the current study we did not follow the survival and distribution of the transplanted cells. However, in our previous experiments we used PKH-26 and feridex (iron nanoparticles) to follow the MSC-NTF cells after intrastriatal transplantation in rats. We found that only a small percentage of the pre-labeled cells migrated and survived. Nevertheless, the procedure was found to be efficient in showing neuroprotection in models of Parkinson and Huntington diseases [2,3]. As we reported, six weeks after intrastriatal transplantation, about one third of the cells were attracted by macrophages, as indicated by CD68 antibodies. Similarly, in another study we detected small amounts of transplanted MSC-NTF cells in the transplanted muscles, after sciatic nerve injury [5]. Further studies will be needed to determine whether MSC-NTF can survive in muscle tissue for an extended period of time.

## Conclusions

Based on the results of this study, we conclude that cryopreserved MSC-NTF cells known as NurOwn™, when injected by either one, two or three repeated IM injections interspaced by four weeks in male and female C57BL/6 J mice, did not generate any significant systemic toxic effects. Histopathological evaluation supports this conclusion and points exclusively to a local effect of minimal severity in the injected muscles.

## Abbreviations

MSC: Mesenchymal stem cells; NTF: Neurotrophic factors; MSC-NTF cells: Neurotrophic factor-secreting MSC; GDNF: Glial Cell-derived neurotrophic factor; BDNF: Brain-derived neurotrophic factor; VEGF: Vascular endothelial growth factor; HGF: Hepatocyte growth factor; IT: Intrathecal administration; IM: Intramuscular; cAMP: Dibutyryl cyclic AMP; hbFGF: Human basic fibroblast growth factor; PDGF-AA: Human platelet derived growth factor; DMSO: Dimethyl sulfoxide; MHC: Major histocompatibility complex.

## Competing interests

N Abramov and Y Gothelf are employees of BrainStorm Cell Therapeutics. D Offen is Scientific Advisor to BrainStorm Cell Therapeutics.

## Authors’ contributions

YG and NA developed the cryopreservation process. AH, DO and YG participated in the design of the study and DO wrote the paper. All authors read and approved the final manuscript.
